# The Convergence of Alpha-Synuclein, Mitochondrial, and Lysosomal Pathways in Vulnerability of Midbrain Dopaminergic Neurons in Parkinson’s Disease

**DOI:** 10.3389/fcell.2020.580634

**Published:** 2020-12-14

**Authors:** Georgia Minakaki, Dimitri Krainc, Lena F. Burbulla

**Affiliations:** Department of Neurology, Northwestern University Feinberg School of Medicine, Chicago, IL, United States

**Keywords:** alpha-synuclein, calcium, dopamine, mitochondria, iron, Parkinson’s disease, synapse

## Abstract

Parkinson’s disease (PD) is the second most common neurodegenerative disease, characterized by progressive bradykinesia, rigidity, resting tremor, and gait impairment, as well as a spectrum of non-motor symptoms including autonomic and cognitive dysfunction. The cardinal motor symptoms of PD stem from the loss of *substantia nigra* (SN) dopaminergic (DAergic) neurons, and it remains unclear why SN DAergic neurons are preferentially lost in PD. However, recent identification of several genetic PD forms suggests that mitochondrial and lysosomal dysfunctions play important roles in the degeneration of midbrain dopamine (DA) neurons. In this review, we discuss the interplay of cell-autonomous mechanisms linked to DAergic neuron vulnerability and alpha-synuclein homeostasis. Emerging studies highlight a deleterious feedback cycle, with oxidative stress, altered DA metabolism, dysfunctional lysosomes, and pathological alpha-synuclein species representing key events in the pathogenesis of PD. We also discuss the interactions of alpha-synuclein with toxic DA metabolites, as well as the biochemical links between intracellular iron, calcium, and alpha-synuclein accumulation. We suggest that targeting multiple pathways, rather than individual processes, will be important for developing disease-modifying therapies. In this context, we focus on current translational efforts specifically targeting lysosomal function, as well as oxidative stress *via* calcium and iron modulation. These efforts could have therapeutic benefits for the broader population of sporadic PD and related synucleinopathies.

## Genetic Links to Pathways of Parkinson’s Disease Vulnerability

Parkinson’s disease (PD) is the most common movement disorder, with a late onset for the majority of cases, affecting 1–2% of the population ≥65 years old (Poewe et al., 2017). The cardinal motor symptoms of PD are bradykinesia, rigidity, and resting tremor, leading to gait impairment and dynamic postural control dysfunction that compromise the quality of life for patients (Kalia and Lang, 2015). The onset of PD motor deficits is largely attributed to dopamine (DA) deficiency within the basal ganglia, due to the deterioration of *substantia nigra pars compacta* (SNpc) neurons (Damier et al., 1999). The hallmark cellular pathology of most but not all PD cases consists of intraneuronal eosinophilic inclusions, namely, Lewy bodies and Lewy neurites, depending on their localization (Goedert et al., 2013; Surmeier et al., 2017). Despite being linked to PD, the timing and dynamics of the formation of Lewy bodies/neurites over the course of the disease remain enigmatic. Biochemical and ultrastructural evidence suggests that aggregated/fibrillar alpha-synuclein (aSyn), encoded by *SNCA*, is a defining feature of Lewy pathology in PD and Dementia with Lewy bodies, as well as intraglial inclusions in Multiple System Atrophy, diseases referred to as alpha-synucleinopathies (Spillantini et al., 1997; Braak et al., 2003; Goedert et al., 2013; Lashuel, 2020). Lipids and membranous organelles have also been documented in Lewy body structures (Lashuel, 2020). A recent ultrastructural study using correlative and multi-imaging approaches demonstrated that aSyn-immunopositive Lewy inclusions also consist of lipid membranes and apparently damaged mitochondrial and lysosomal organelles with variable spatial organization (Shahmoradian et al., 2019). This study is contemporary with the increasing genetic evidence for the contribution of mitochondrial and lysosomal processing pathways, converging with aSyn homeostasis in PD pathogenesis (Mazzulli et al., 2011; Burbulla et al., 2017; Wong and Krainc, 2017). Thus, elucidating the interactions between aSyn and mitochondrial, lysosomal, and lipid homeostasis pathways in SNpc dopaminergic (DAergic) neurons will help understand PD progression and may be beneficial for the discovery of new therapeutic targets to develop novel therapeutic strategies.

PD is a predominantly sporadic disease with aging representing the greatest risk factor, whereas up to 10% of cases occur in a familial manner with both autosomal dominant and recessive transmission (Hernandez et al., 2016). It is also likely that genetic as well as environmental causes contribute to both familial and sporadic PD, albeit to different extents (Singleton et al., 2013). *SNCA* was the first gene with mutations reported to cause autosomal dominant PD (A53T missense mutation), and to date, additional five missense *SNCA* mutations have been identified (A30P, A53E, E46K, G51D, and H50Q) (Polymeropoulos et al., 1997; Kruger et al., 1998; Zarranz et al., 2004; Appel-Cresswell et al., 2013; Lesage et al., 2013; Proukakis et al., 2013; Pasanen et al., 2014). Other genes have also been considered causal in autosomal dominant PD (including *LRRK2*, *VPS35*). Genes associated with autosomal recessive inheritance patterns are implicated in typical, early onset disease (*PARKIN*, *PINK1*, *DJ-1*, *FBXO7*) (Blauwendraat et al., 2020) and point toward functional implications for, but not limited to, the mitochondrial clearance pathway. Further atypical forms of parkinsonism, with juvenile onsets, include genes implicated in lysosomal function (*ATP13A2*) and synaptic vesicle endocytosis (SVE) pathway (*DNAJC6* and *SYNJ1*). Among several other genes, *LRRK2*, *SNCA*, *GBA1*, *CTSB*, and *SH3GL2* are associated with PD risk, as validated in the largest meta-analysis of genome-wide association studies (GWASs) to date (Nalls et al., 2019). Collectively, genetic evidence suggests that in both sporadic and familial PD, mitochondrial, endolysosomal, and synaptic vesicle homeostasis are compromised (Hardy, 2010; Burbulla et al., 2017; Nalls et al., 2019; Nguyen et al., 2019).

Understanding the molecular mechanisms underlying the preferential susceptibility of SNpc DAergic neurons in PD remains a major focus of research. Because most currently available PD animal models do not manifest key pathogenic phenotypes of human pathology (Dawson et al., 2018), it has been difficult to study these mechanisms *in vivo*, representing a major caveat in modeling the disease. Furthermore, the increasing appreciation of multiple genetic “hits” contributing to PD raises the importance of addressing mechanistic interactions and potential pathway intersections using models representative of patient genetic heterogeneity. A significant development in this direction has been the establishment of human induced pluripotent stem cell (iPSC) technology allowing for long-term cultures of highly enriched patient-derived DAergic neurons (Kriks et al., 2011; Mazzulli et al., 2011; Burbulla et al., 2017). Using this technology, neurons from patients with various PD genetic backgrounds have been studied addressing oxidative stress levels, mitochondrial function, and lysosomal and calcium homeostasis, whereas pathological aSyn remains a central aspect of investigation. aSyn plays a multifaceted pathological role in PD and related synucleinopathies, with aSyn-dependent toxicity impacting various cellular pathways (Wong and Krainc, 2017). Here, we focus on studies providing mechanistic insight into how interactions between aSyn, mitochondrial, endolysosomal, and synaptic pathways may be linked to DAergic vulnerability in PD. When feasible, emphasis is given to evidence derived from primary neurons and iPSC-derived DAergic neuronal models.

## Alpha-Synuclein and Major Determinants of PD Dopaminergic Vulnerability

The neuromelanin-containing SNpc neurons, sending DAergic projections to the dorsal striatum, are preferentially vulnerable in PD (Hirsch et al., 1988; Damier et al., 1999). A major concept is that degeneration is initiated at DAergic terminals, followed by the soma (Cheng et al., 2010; Grosch et al., 2016). Quantification of DAergic phenotype deterioration using human *post mortem* tissue estimated that clinical PD manifestations are paralleled by ∼50% reduction of putaminal expression of tyrosine hydroxylase (TH), the rate limiting enzyme in DA production (Kordower et al., 2013). Interestingly, while the putaminal TH and DA transporter (DAT) expressions are lost within 4 years of diagnosis, a small number of residual TH and neuromelanin-containing SNpc neurons may persist even after 27 years (Kordower et al., 2013).

A major focus is placed on the distinct anatomical and functional features of SNpc DAergic neurons, linked to cell-autonomous pathways of vulnerability (Surmeier et al., 2017; Wong et al., 2019). Firstly, single-cell tracing followed by digital reconstruction indicated that rat SNpc DAergic neurons possess widely spread and highly dense axonal arborization within the striatum (Matsuda et al., 2009). Such an architecture can give rise to numerous synaptic connections and leads to high bioenergetic demands (Matsuda et al., 2009; Bolam and Pissadaki, 2012). Assuming a similar nigrostriatal system architecture, human SNpc DAergic neurons were estimated to form even more synaptic connections, linked to even higher bioenergetic demands (Bolam and Pissadaki, 2012). Based on computational modeling, larger size and axonal arbor complexity significantly increase the energetic cost for maintaining axon potential propagation and recovering the resting membrane potential (Pissadaki and Bolam, 2013). Secondly, free cytosolic DA and DA metabolites are prone to oxidation, generating reactive quinones that can be neurotoxic and able to readily modify proteins causing protein aggregation (Sulzer and Zecca, 1999). Thirdly, midbrain DAergic neurons are autonomous pacemakers, involving spontaneous action potentials firing in a regular rhythmic manner (Bean, 2007). The accompanying entry of calcium during repolarization, which supports pacemaking, DA synthesis, and ATP production, also increases mitochondrial oxidative stress (Bean, 2007). Notably, while SNpc neurons are lost in PD, the neighboring ventral tegmental area (VTA) DAergic neurons are more resilient—a fact that has been partly attributed to the less extensive arborization and synaptic network, lower somatodendritic density of calcium channels (L-type), and higher endogenous expression of calcium buffering proteins (Surmeier and Schumacker, 2013). In addition, DA metabolism may be different between SNpc and VTA neurons, possibly leading to higher levels of toxic intermediates of DA catabolism in PD (Galter et al., 2003), as will be discussed in more detail.

### Alpha-Synuclein and the Synaptic Compartment

**FIGURE 1:**
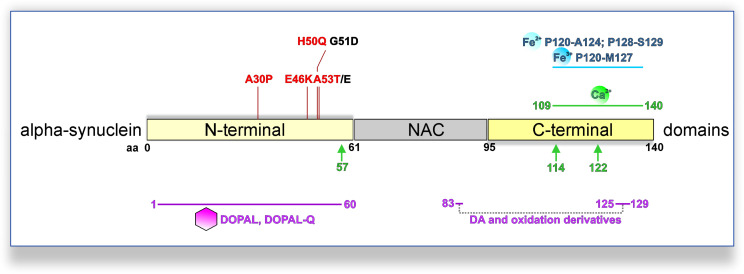
PD-linked *SNCA* mutations and interactions with DA metabolites, calcium, or iron promoting pathological aSyn oligomerization and/or aggregation. PD-linked *SNCA* missense mutations that increase the oligomerization and/or fibrillization of aSyn *in vitro* are shown in red (otherwise black). DA and oxidation derivatives bind non-specifically to the C-terminus (aa 125–129), further stabilized by long-range electrostatic interactions with E83 of the NAC region (intermittent gray line). Also, DOPAL/DOPAL-Q adducts with N-terminal aSyn lysines are formed at the 1–60 domains (purple line). Fe^2+^ (ferrous) and Fe^3+^ (ferric) iron bind to adjacent regions at the C-terminus of aSyn (blue line; Fe^2+^: aa P120-A124 and P128-S129; Fe^3+^: aa P120-M127). Moreover, a calcium-binding motif has been mapped to the C-terminus of aSyn (green line; aa 109–140). Calcium also promotes calpain I-mediated cleavage of aSyn, with three major cleavage sites depicted (green arrows; monomeric aSyn: after aa 57; fibrillar aSyn: after aa 114 and 122).

Dysregulation of aSyn homeostasis has detrimental consequences for many pathways implicated in PD DAergic system vulnerability. aSyn is a 140 amino acid protein ubiquitously expressed in the central nervous system, predominately localized at the presynaptic compartment (Maroteaux et al., 1988; Iwai et al., 1995). The primary amino acid sequence can be divided into three domains (Figure 1): the N-terminal domain (amino acid 1–60), the central domain also termed non-amyloid component of Aβ plaques (NAC; 61–95), and the C-terminal domain (96–140). The N-terminal domain mediates the association of aSyn with anionic phospholipids and shows preferential binding to smaller (∼45 μm diameter) vesicles (Middleton and Rhoades, 2010). Accordingly, biochemical and ultrastructural analyses indicated that aSyn associates with the membrane of synaptic vesicles (Maroteaux et al., 1988; Emmanouilidou et al., 2016; Vargas et al., 2017). aSyn oligomers are considered as primary pathogenic species in PD and related synucleinopathies. Interaction of aSyn oligomers with synaptic vesicle-like membranes demonstrated that N-terminus-mediated binding to membranes allows the rigid oligomeric core to be inserted into membranes and disrupt their integrity (Fusco et al., 2017). Interestingly, all familial PD *SNCA* mutations are localized in the N-terminal region of aSyn (A30P, A53E/T, E46K, G51D, and H50Q) (Polymeropoulos et al., 1997; Kruger et al., 1998; Zarranz et al., 2004; Appel-Cresswell et al., 2013; Lesage et al., 2013; Proukakis et al., 2013; Pasanen et al., 2014), and with the exception of G51D and A53E potentiate aSyn oligomerization and/or fibrillization *in vitro*, but exhibit differential propensity of forming inclusions within cells (Conway et al., 2000; Fares et al., 2014; Lazaro et al., 2014, Lázaro et al., 2016). In particular, a physiological role of aSyn in neurotransmission and synaptic vesicle trafficking has been demonstrated (Wong and Krainc, 2017). aSyn interacts with the vesicle-associated membrane protein (VAMP) 2 and promotes SNARE (Soluble N-ethylmaleimide-sensitive factor Attachment Protein Receptor) complex assembly (Burre et al., 2010), which enables neurotransmitter release *via* synaptic vesicle exocytosis. However, germline *Snca*-knockout mouse studies suggested that aSyn is not essential for basal neurotransmission in DAergic neurons, but rather plays a regulatory role in activity-dependent DA release (Abeliovich et al., 2000; Cabin et al., 2002; Chandra et al., 2004). Nevertheless, the specific physiological function of aSyn within the central nervous system requires further elucidation.

**FIGURE 2:**
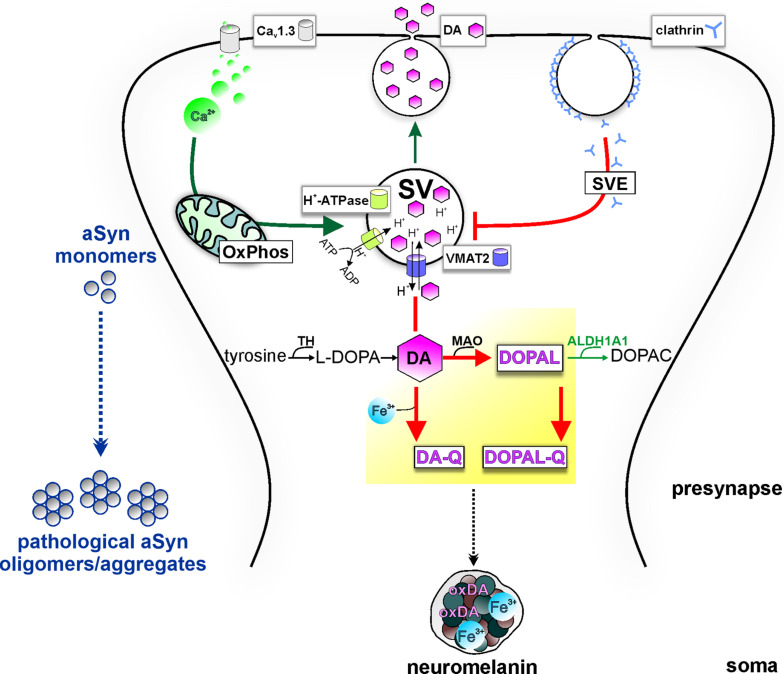
Potential model of the intersection of PD vulnerability pathways with DA metabolism at presynapse, promoting aSyn aggregation. Pre-synaptic calcium influx (*via* Ca_v_1.3 calcium channels) drives mitochondrial oxidative phosphorylation (OxPhos), energetically supporting DA sequestration (thick green arrow) and consecutively neurotransmission. Synaptic vesicles (SVs) are loaded with DA neurotransmitter by VMAT-2, which transports cytosolic DA into the SV in exchange of H^+^ provided by the action of the H^+^-ATPase. Following release, synaptic vesicle endocytosis (SVE) replenishes SVs; however, PD-linked deficits in this pathway can limit the SV availability (thick red line). Because of ongoing DA biosynthesis (TH-catalyzed conversion of tyrosine to L-DOPA), impairment of DA sequestration into SVs leads to the accumulation of DA in the cytosol (thick red line). Subsequent build-up of oxidized DA metabolites (yellow highlight) in the cytosol disturbs aSyn homeostasis and leads to neurotoxicity. Specifically, cytosolic DA oxidation, catalyzed by iron (Fe^3+^), can lead to increased oxidized DA derivatives (oxDA), as well as increased levels of the neurotoxic metabolite DOPAL, catalyzed by MAO, further oxidized to DOPAL-Q (thick red arrows). aSyn oligomerization by DOPAL/-Q entails formation of adducts, whereas binding of DA-Q, calcium, and iron also promote the formation of aSyn oligomers/aggregates. Neuronal detoxification from DOPAL is mediated by ALDH1A1, which catalyzes its conversion to the non-toxic metabolite DOPAC (thin green arrow). Moreover, DA-Q and iron are removed from the cytosol over time by sequestration to the dark pigment neuromelanin at the soma (black intermittent arrow), enclosed within membranes, which can be neuroprotective under physiological conditions.

Aggregated pathological forms of aSyn at both presynaptic terminals (Kramer and Schulz-Schaeffer, 2007) and along the axon (Braak et al., 1999) have been described in *post mortem* patient tissue, implicating aSyn pathology in synaptic deterioration. *SNCA* overexpression in transgenic mouse models has been linked to nigrostriatal terminal loss (Masliah et al., 2000), DA release deficits, and changes in synaptic vesicle clustering (Janezic et al., 2013). aSyn overexpression in ventral midbrain primary neuron cultures was linked to inhibition of the SVE pathway, attributed to a reduced recycling vesicle pool and defective reclustering after endocytosis (Nemani et al., 2010). Interestingly, SVE coordinates the replenishment of synaptic vesicles following neurotransmission and has emerged as a pathway of PD vulnerability, with *SYNJ1* (synaptojanin 1) and *DNAJC6* (auxilin) implicated as causal (Blauwendraat et al., 2020) or *SH3GL2* (endophilin A1) as risk factor in PD (Nalls et al., 2019; Nguyen et al., 2019). Briefly, SVE begins with the invagination of a new clathrin-coated vesicle from the plasma membrane, with the participation of endophilin A1 and synaptojanin 1. Subsequently, auxilin-mediated removal of the clathrin lattice allows for the refilling with neurotransmitters, such as DA. *LRRK2* mutations, leading to hyperactivation of LRRK2 kinase, are linked to SVE deficits since endophilin A1, synaptojanin 1, and auxilin are LRRK2 phosphorylation substrates (Matta et al., 2012; Islam et al., 2016; Nguyen and Krainc, 2018). Using *LRRK2* PD iPSC-derived DAergic neurons, it was demonstrated that increased phosphorylation in the clathrin-binding domain of auxilin (S627) by LRRK2 impaired clathrin uncoating (Nguyen and Krainc, 2018). This mechanism was linked to the reduced density of synaptic vesicles at terminals, which concurred with oxidized DA accumulation and increased aSyn levels (Nguyen and Krainc, 2018). Based on this evidence, a synergistic effect of LRRK2 and aSyn on SVE could lead to decreased DA packaging into synaptic vesicles, thus contributing to PD vulnerability (Figure 2). In addition to synaptic deficits, axonopathy has also been reported in iPSC-derived neuronal cultures from A53T *SNCA* patients (Kouroupi et al., 2017). These cultures showed swollen varicosities immunopositive for aSyn, which were improved by small molecules targeting aSyn aggregation (Kouroupi et al., 2017).

### Alpha-Synuclein and Mitochondria

Mitochondria are present in the synaptic compartment (Graham et al., 2017; Reeve et al., 2018) and crucial for maintaining synaptic activity and neuronal homeostasis by ATP production, calcium storage, and lipid metabolism (Nunnari and Suomalainen, 2012). Genetic evidence suggests that the pathways of mitochondrial quality control (*PARKIN*, *PINK1*, *DJ-1*, *FBOX7*) and mitochondrial fusion/fission dynamics (*OPA1*) are implicated in PD and parkinsonism (Carelli et al., 2015; Lynch et al., 2017). Further biochemical studies showed mitochondrial respiratory chain complex I deficiency in PD SN tissue (Schapira et al., 1990). Hampering local energy support of DAergic synapses due to mitochondrial dysfunction could significantly contribute to progressive loss of nigrostriatal connections. Supporting this notion, *post mortem* immunofluorescence analysis showing putaminal loss of DAergic synapses in PD patients reported higher mitochondrial marker per axon volume (Reeve et al., 2018), which may reflect compensatory increased trafficking for energy support.

Aberrant aSyn–mitochondria interactions resulting in oxidative/nitrosative stress, as well as mitochondrial trafficking and respiration defects, can render DAergic synapses vulnerable. Application of recombinant oligomeric aSyn species to neuroblastoma cells and primary cortical neurons disrupted plasma membrane integrity, significantly increased intracellular reactive oxygen species (ROS), and reduced mitochondrial activity (Fusco et al., 2017). Formation of pathological oligomeric aSyn species within neurons, resulting from wild-type aSyn overexpression or expression of mutant aSyn, has been associated with reduced anterograde mitochondria trafficking (Prots et al., 2018) and mitochondrial fragmentation (Kamp et al., 2010; Nakamura et al., 2011; Cali et al., 2012; Guardia-Laguarta et al., 2014; Menges et al., 2017). Mitochondrial transport defects were paralleled by reduced axonal fiber density and decreased synaptic robustness (Prots et al., 2018). Microfluidic cultures of iPSC-derived neurons modeling DAergic-medium spiny neuron synapse showed that PD *OPA1* mutant iPSC-derived DAergic neurons had impaired anterograde mitochondrial trafficking leading to progressive synaptic loss (Iannielli et al., 2019). Mitochondria within *OPA1* mutant neurons exhibited a fragmented phenotype, and it is not known whether fragmentation may *per se* reduce mitochondrial trafficking. If so, the role of aSyn in the regulation of mitochondrial dynamics could be crucial, and further investigation is required. Direct interaction of both wild-type and mutant (A53T, A30P) aSyn has been shown at mitochondria-associated membranes (MAMs) (Guardia-Laguarta et al., 2014), domains of endoplasmic reticulum (ER)–mitochondria contact, relevant for the exchange of ions and lipids. The aSyn–MAMs interaction has been suggested to enhance mitochondrial calcium uptake from the ER (Cali et al., 2012), which could in turn affect ATP production and oxidative stress. Furthermore, aSyn accumulation in association with mitochondria can reduce complex I activity and elevate mitochondrial ROS production in DAergic neurons (Devi et al., 2008; Chinta et al., 2010). Proximity ligation assays in human *post mortem* PD and rat SN tissue indicated binding of aSyn to TOM20 (translocase of the outer membrane receptor 20), which can be a crucial interaction underlying aSyn-dependent complex I deficiency and oxidative stress (Di Maio et al., 2016). Specifically, it was shown that *via* TOM20 binding, oligomeric, DA-modified, and phosphorylated (S129E phosphomimetic mutant) aSyn species could hamper the mitochondrial import of the complex I subunit Ndufs3, linked to lower basal respiration and oxidative damage (Di Maio et al., 2016). The aSyn–TOM20 interaction has also been demonstrated in iPSC-derived DAergic neurons, possibly contributing to mitochondrial dysfunction in A53T and *SNCA* triplication PD neurons (Zambon et al., 2019). Compared with isogenic controls, A53T *SNCA* pluripotent stem cell-derived DAergic neurons showed exacerbated ROS/reactive nitrogen species (RNS) production and higher susceptibility upon exposure to mitochondrial toxins (Ryan et al., 2013). Furthermore, gene expression analysis in sporadic PD SNpc tissue, and comparison between A53T *SNCA* and isogenic pluripotent stem cell-derived DAergic neurons, has linked axodendritic pathology to an impaired antioxidative stress response (Czaniecki et al., 2019).

### Alpha-Synuclein and Dopamine

DA signaling and metabolism are regulated by the coordinated actions of enzymes and transporters responsible for DA synthesis, packaging into vesicles and re-uptake following release, or for degradation of cytosolic DA. Sequestration or degradation is particularly important as the accumulation of DA in the cytosol generates ROS and oxidizes DA by-products (Figure 2). TH converts dietary tyrosine to L-dihydroxyphenylalanine (L-DOPA), which is ultimately converted to DA by aromatic amino acid decarboxylase (Molinoff and Axelrod, 1971). Following synthesis, the vesicular monoamine transporter (VMAT)-2 uses ATP-driven vesicular electrochemical gradient to package cytosolic DA into vesicles along the axon and pre-synapses, but also into tubular structures at the somatodendritic compartment (Li et al., 2005; Figure 2). Neurotransmission is terminated by high-affinity DA reuptake through DAT at the pre-synapse, where DA can be recycled for exocytosis (Figure 2). At this stage, deficits in the SVE pathway, as reported in *LRRK2* PD iPSC-derived DAergic neurons (Nguyen and Krainc, 2018) among other cellular and *in vivo* models (Nguyen et al., 2019), can lead to DA accumulation in the cytosol. Preventing the potentially neurotoxic effects of cytosolic build-up, the main degradation route begins with DA oxidative deamination to 3,4-dihydroxyphenylacetaldehyde (DOPAL), concurring with H_2_O_2_ (hydrogen peroxide) generation, by the outer mitochondrial membrane monoamine oxidase (MAO) (Masato et al., 2019). Furthermore, the DOPAL metabolite is potentially toxic for DAergic cells and is by far more potent than DA in both oligomerizing and forming quinone protein adducts with aSyn (Burke et al., 2008; Goldstein et al., 2012; Jinsmaa et al., 2020). Therefore, the route of conversion to the corresponding non-toxic 3,4-dihydroxyphenylacetic acid (DOPAC), catalyzed by aldehyde dehydrogenase ALDH1A1 (Masato et al., 2019), is an important detoxification mechanism (Figure 2). Subsequently, DOPAC is degraded mainly to homovanillic acid by catechol−O−methyltransferase (Masato et al., 2019). Dysregulated DA metabolism has been linked to SNpc vulnerability in PD since *post mortem* analyses showed decreased *ALDH1A1* mRNA in PD SNpc but not VTA (Liu et al., 2014) and higher DOPAL:DOPAC and DOPAL:DA ratios in PD putamen (Goldstein et al., 2011, 2013). In addition, theoretical indices derived from DA/metabolites relative ratios have suggested that both reduced vesicular DA uptake and ALDH1A1 activity accounted for DOPAL accumulation in PD putamen (Goldstein et al., 2013). Quantitative proteomics and transcriptomics highlighted mitochondrial and lysosomal dysregulation and differential vulnerability of DAergic neurons derived from several isogenic PD gene knockout pluripotent stem cell lines (Ahfeldt et al., 2020).

**FIGURE 3:**
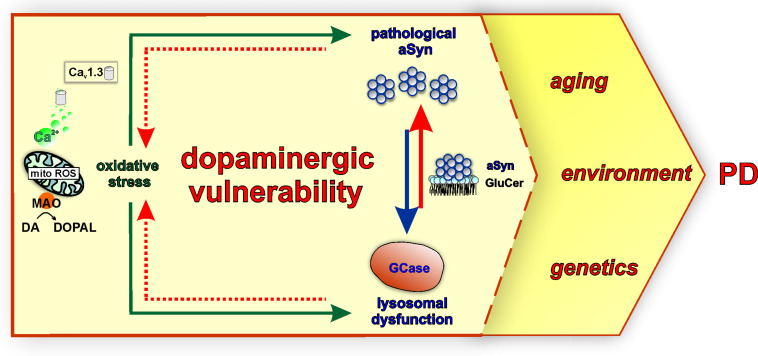
Mitochondrial oxidative stress, lysosomal dysfunction, and pathological aSyn are major converging pathways in the vulnerability of SNpc DAergic neurons in PD. Calcium influx *via* Ca_v_1.3 channels during pacemaking and the metabolism of DA to DOPAL by mitochondrially-anchored MAO support mitochondrial energy production under physiological conditions. However, these mechanisms could also represent a dual source of sustained mitochondrial oxidative stress (mito ROS) over a long period, contributing to PD SNpc vulnerability. Moreover, DA metabolism and neurotoxic DA metabolites including DA/DOPAL quinones exacerbate oxidative stress and are linked to pathological aSyn modifications and GCase inhibition (green arrows). In turn, GCase is reciprocally linked to pathological aSyn *via* its lipid substrate glucosylceramide (GluCer). GCase deficiency increases GluCer levels, therefore stabilizing oligomeric aSyn intermediates (red arrow), whereas pathological aSyn inhibits GCase lysosomal activity or trafficking (blue arrow). As part of this vicious feedback cycle, both GCase and pathological aSyn contribute to oxidative stress (intermittent red arrow) *via* intersecting with DA metabolism. These major pathways may render SNpc DAergic neurons more vulnerable to the influence of PD-linked factors, such as aging, environment, and genetics.

Mechanistically, dysregulated DA metabolism has emerged as a significant SNpc vulnerability pathway, converging with mitochondrial oxidative stress, with implications for both lysosomal dysfunction and aSyn pathology (Figure 3). In line with this, iPSC-derived DAergic neurons from an *SNCA* duplication patient showed higher aSyn aggregation/phosphorylation as well as higher ROS/RNS levels than cortical neurons derived from the identical iPSC line, although the underlying mechanisms require elucidation (Brazdis et al., 2020). Cytosolic DA can be non-enzymatically converted to reactive DA-*o*-quinone (DA-Q), catalyzed by Fe^3+^ (ferric) iron (Zucca et al., 2017). Additional quinones and semiquinones are sequentially formed, thus aggravating oxidative stress (Zucca et al., 2017). Importantly, accumulation of oxidized DA/DA-Q represents a key phenotype developing over time in human iPSC-derived DAergic neurons from genetic PD forms related to mitochondrial (*PARKIN*, *PINK1*, *DJ-1*) and endolysosomal pathways (*LRRK2*, *GBA1*) or sporadic PD (Burbulla et al., 2017, 2019; Ysselstein et al., 2019; Laperle et al., 2020). DA promotes the accumulation of pathological aSyn protofibril intermediates, involving oxidation to DA-Q (Conway et al., 2001). Moreover, elevation of DA in mouse SN neurons by lentiviral overexpression of catalytically hyperactive TH promoted the formation of pathological aSyn oligomers and accelerated the DAergic deterioration of A53T aSyn overexpressing mice (Mor et al., 2017). Another potential DAergic vulnerability mechanism linked to the aSyn–DA interaction is the impaired lysosomal degradation of proteins cleared *via* chaperone-mediated autophagy. DA-/autoxidized DA-modified aSyn was shown to bind to the lysosomal membrane, but was not efficiently taken up and degraded within the lumen (Martinez-Vicente et al., 2008). In addition, elevation of cytosolic DA in mouse primary ventral midbrain neurons by L-DOPA administration inhibited protein degradation *via* chaperone-mediated autophagy in wild-type but not aSyn null neurons (Martinez-Vicente et al., 2008). Based on molecular dynamic simulations, DA and oxidation derivatives non-specifically bind to the C-terminus of aSyn (aa 125–129), further stabilized by long-range electrostatic interactions with the NAC region (aa E83) (Herrera et al., 2008; Figure 1). An additional mechanism of DA-related toxicity is the modification of proteins by DA-Q, with adducts formed by the catechol moiety reacting with thiols of cysteines (Masato et al., 2019). Two examples of DA-Q modified proteins are parkin and the lysosomal glucocerebrosidase (GCase). In neuronal cell lines, DA-Q-modified parkin showed lower E3 ubiquitin ligase activity and decreased solubility (LaVoie et al., 2005). Moreover, catechol-modified parkin was detected in human control SNpc (LaVoie et al., 2005). In addition, DA-Q modification of cysteines at the GCase catalytic site leads to decreased lysosomal GCase activity in iPSC-derived DAergic neurons, thus contributing to lysosomal dysfunction (Burbulla et al., 2017). The primary DA metabolite DOPAL is highly neurotoxic *in vitro* and *in vivo*. Like DA, DOPAL is auto-oxidized to quinones (DOPAL-Q) with concomitant generation of ROS. In addition to the reported general increase in cellular oxidative stress linked to DOPAL and other DA oxidation derivatives, mitochondria can also be specifically impacted. Use of H_2_O_2_-sensitive genetically encoded thiol redox sensors indicated that short-term L-DOPA treatment of DAergic neurons induces MAO activity for catabolism of DA to DOPAL and concomitantly increases oxidative stress specifically within axonal mitochondria (Graves et al., 2020). Although this mechanism was shown to be physiologically relevant for supporting ATP production (Graves et al., 2020), it may also represent an additional source of mitochondrial stress in the PD *milieu*, accentuating DAergic neuron axonal/synaptic vulnerability. DOPAL is also linked to aSyn pathology, with injection into rat SN resulting in high molecular weight aSyn species and loss of TH^+^ neurons (Burke et al., 2008). DOPAL/DOPAL-Q modify aSyn *via* the formation of adducts between DOPAL/DOPAL-Q aldehyde moiety and N-terminal aSyn lysines (domain of aa 1–60), thus increasing aSyn oligomerization (Follmer et al., 2015). aSyn modification by DOPAL could lead to loss of aSyn function at the presynapse, since it was shown that DOPAL-modified aSyn has reduced affinity for synaptic vesicle-like membranes (Follmer et al., 2015), and that DOPAL-aSyn oligomers have been shown to permeabilize lipid membranes leading to DA leak (Plotegher et al., 2017). This leads to a vicious cycle of cytosolic DA/DOPAL accumulation, oxidative stress, toxic protein modifications promoting pathological aSyn, and lysosomal dysfunction *via* GCase. Addressing DOPAL build-up mechanisms in detail using iPSC-derived DAergic neurons will provide further insight into how DA release/metabolism equilibrium may be altered in PD. Importantly, such studies could open new venues for DA detoxification approaches, as an early intervention in PD.

Oxidized DA and derivatives also constitute the basis for the formation of neuromelanin, a dark polymeric pigment, which accumulates over a lifetime in human SN neurons and is considered as detoxifying under physiological conditions (Zucca et al., 2018). Neuromelanin has been observed in *DJ-1* PD iPSC-derived DAergic neurons (Burbulla et al., 2017) and iPSC-derived midbrain-like organoids treated with DA or L-DOPA (Jo et al., 2016). Electron microscopy of human SN revealed neuromelanin-containing organelles enveloping dark proteinaceous granular aggregates and lipid bodies (Zucca et al., 2018). Moreover, biochemical analyses indicated a high representation of endolysosomal pathway proteins, as well as higher molecular weight aSyn species, within neuromelanin organelles (Zucca et al., 2018). In addition to proteins and lipids, metals, such as Fe^3+^ (ferric) iron, which catalyzes DA oxidation, are bound to neuromelanin. Indeed, neuromelanin’s composition is indicative of the intersection between DA metabolism and aSyn-related pathways, with iron playing a significant modulatory role. Iron is enriched in the basal ganglia and SN (Hallgren and Sourander, 1958), whereas specifically in the putamen, iron levels increase with age reaching maximum concentration at 50–60 years (Hallgren and Sourander, 1958). Interestingly, another group of diseases shares the feature of high brain iron in the basal ganglia and progressive neurodegeneration with PD. This is the very rare and heterogeneous group of neurodegeneration with brain iron accumulation (NBIA) disorders, characterized by young onset and progressive extrapyramidal dysfunction (Neumann et al., 2000). Furthermore, certain NBIA cases show extensive Lewy pathology in the SN, and specifically SNpc, as well as the cerebral cortex (Arawaka et al., 1998; Neumann et al., 2000; Tofaris et al., 2007). Importantly, accumulation of iron in the SN of PD patients has been reported by several studies (Dexter et al., 1991; Griffiths et al., 1999; Graham et al., 2000). It is also possible that regulation of iron metabolism in the periphery is altered in PD as in the case of ceruloplasmin, a liver-synthesized enzyme that converts Fe^2+^ (ferrous iron) to Fe^3+^, thus facilitating peripheral iron transportation. Reduced ceruloplasmin activity has been reported in the SN and biofluids of some PD patients, and missense variations in the ceruloplasmin-encoding gene showed a possible link with SN iron deposition in few PD patients (Ward et al., 2014). Iron can contribute to the pathways of oxidative stress and aSyn pathology (Figure 2). After reduction from the Fe^3+^ to the Fe^2+^ state, iron can participate in Fenton reactions that liberate radicals and can result in neurotoxic protein oxidation or lipid peroxidation (Halliwell and Gutteridge, 1984; Winterbourn, 1995). Furthermore, iron binds to the C-terminus of aSyn (Figure 1; Fe^2+^: aa P120-A124 and P128-S129; Fe^3+^: aa P120-M127), increased by S129 phosphorylation (Lu et al., 2011), a post-translational modification identified in Lewy bodies (Fujiwara et al., 2002). The aSyn–iron interaction is potentially toxic, since iron alone or in concert with DOPAL promotes aSyn oligomerization (Hashimoto et al., 1999; Uversky et al., 2001; Jinsmaa et al., 2014). Conversely, aSyn can enhance iron toxicity *via* conversion of Fe^3+^ to Fe^2+^, leading to higher DOPAL susceptibility in neuronal cells (McDowall et al., 2017). Such activity has also been demonstrated *in vivo* following viral overexpression of human aSyn in the rat SN, leading to TH neuron loss (McDowall et al., 2017). Consequently, aSyn–iron interactions can aggravate oxidative stress and contribute to DAergic neuron vulnerability.

Mitochondrial antioxidants including N-acetylcysteine (precursor to glutathione synthesis) have also been shown to prevent the accumulation of oxidized DA and aSyn in iPSC-derived DAergic cultures (Burbulla et al., 2017). Furthermore, antioxidant polyamines have recently been identified as substrates of the PD-linked lysosomal ATPase ATP13A2 (van Veen et al., 2020). Although the mechanisms are unclear, polyamine metabolism may be altered during aging but also in PD. *Post mortem* examinations have indicated a decrease of polyamines in basal ganglia (Vivó et al., 2001), and in PD, there appears to be a reduced expression of the catabolic enzyme spermidine/spermine N1-acetyltransferase 1 (SAT1) (Lewandowski et al., 2010). Further elucidation of functional interactions between modulators of oxidative stress and PD-linked genes will be important for novel, targeted PD antioxidant therapies.

### Alpha-Synuclein and Calcium

aSyn and calcium homeostasis appear to be reciprocally regulated. Calcium binds to aSyn at a 32-aa C-terminal domain (Figure 1; Nielsen et al., 2001). This interaction promotes aSyn aggregation and can potentiate the interaction between aSyn and synaptic vesicles (Nielsen et al., 2001; Lautenschläger et al., 2018). Calcium can also affect aSyn conformation *via* the calcium-activated protease calpain I. Using recombinant aSyn, one major cleavage site for monomeric (after aa 57) and two additional major sites for fibrillary aSyn (after aa 114 and 122) have been identified (Mishizen-Eberz et al., 2003). *In vivo* calpain inhibition *via* overexpression of the natural inhibitor calpastatin abolished aSyn truncation and could significantly reduce aSyn aggregation in A30P aSyn overexpressing mice (Diepenbroek et al., 2014). Moreover, deficient extracellular vesicle release, a calcium-dependent process partly mediating aSyn secretion (Emmanouilidou et al., 2010; Tsunemi et al., 2014), has been related to progressive aSyn accumulation in *ATP13A2* iPSC-derived DAergic neurons (Tsunemi et al., 2019). Conversely, aSyn can affect intraneuronal calcium homeostasis, with overexpression leading to a sustained elevation of basal calcium levels (Caraveo et al., 2014; Angelova et al., 2016). Specifically, oligomeric aSyn can induce channel formation on artificial membranes leading to irregular calcium influx, which can result in calcium-dependent cellular toxicity (Angelova et al., 2016). Also, *SNCA* triplication iPSC-derived cortical neurons were shown to exhibit abnormal patterns of Ca^2+^ influx upon depolarization, attributed to the integration of oligomeric aSyn species into membranes (Angelova et al., 2020).

In addition, calcium dynamics can contribute to the vulnerability of SNpc neurons, acting in concert with aSyn and DA (Figure 2). Calcium enters DAergic neurons *via* voltage-gated calcium channels (Cav), residing at the axon terminals as well as at the soma and proximal dendrites (Lai and Jan, 2006). The pacemaking activity of SNpc DAergic neurons is accompanied by calcium influx *via* L-type (Ca_v_1.3 subtype) calcium channels, which are activated at relatively negative membrane potential (Surmeier et al., 2017). Measurements using a mitochondrial matrix-targeted indicator showed that L-type channel-mediated calcium influx during pacemaking is linked to mitochondrial calcium influx *via* ER receptors and leads to increased basal levels of mitochondrial oxidative stress (Guzman et al., 2010). This calcium-dependent feed forward mechanism drives oxidative phosphorylation, but also constitutes sustained mitochondrial oxidative stress with a key role in SN DAergic vulnerability (Surmeier et al., 2017). SN mouse primary neurons treated with the Ca_v_1.2/Ca_v_1.3 negative allosteric modulator nimodipine were significantly more resilient to L-DOPA, a treatment that raises cytosolic DA and leads to neurotoxicity (Mosharov et al., 2009). Along this line, application of L-type calcium channel negative allosteric modulators (nimodipine and isradipine) could prevent the exacerbated elevation of cytosolic calcium and reduce susceptibility of SN neurons, as compared with VTA, in a Parkinsonian MPP + (1-methyl-4-phenylpyridinium) neurotoxin model (Lieberman et al., 2017). aSyn knockout neurons did not show elevation of cytosolic calcium following MPP + treatment and were less sensitive to either L-DOPA or MPP+ (Mosharov et al., 2009; Lieberman et al., 2017). Furthermore, *GBA1*-PD and *ATP13A2*-PD iPSC-derived DAergic neurons showed elevated basal calcium levels (Schondorf et al., 2014; Tsunemi et al., 2019), and importantly, inhibition of calcium influx and downstream signaling in long-term *DJ-1* iPSC-derived DAergic neuron cultures could significantly reduce DA oxidation (Burbulla et al., 2017). Collectively, Ca_v_1.3-mediated calcium influx (Guzman et al., 2010) and MAO-mediated DA metabolism (Graves et al., 2020) represent a dual source of sustained oxidative stress in DAergic neuron mitochondria and are reciprocally linked to aSyn and lysosomal homeostasis pathways (Figure 3). Additional calcium channels may play a role in calcium influx-related DAergic toxicity. For instance, single-cell mRNA and protein expression analyses indicated that the Ca_v_2.3 channel subtype is most highly abundant in mature mouse SN DAergic neurons (Benkert et al., 2019). In this study, Ca_v_2.3 channels were shown to contribute to activity-related calcium oscillations, and their knockout protected against MPTP (1-methyl-4-phenyl-1,2,3,6-tetrahydropyridin)-induced SNpc DAergic cell death in mice. Importantly, Ca_v_2.3 expression was readily detected in *GBA1*-PD and healthy control iPSC-derived DAergic neurons (Benkert et al., 2019). These findings corroborate the importance of investigating further molecular players in calcium signaling dysregulation and suggest their contribution to the vulnerability of human DAergic neurons, for revealing new therapeutic targets.

## Lysosomal Dysfunction and Alpha-Synuclein Linked by Glucocerebrosidase

Inefficient clearance *via* the autophagy–lysosome pathway has long been linked to PD (Anglade et al., 1997), and pathological aSyn can interfere with, and compromise, several stages of this pathway (Xilouri et al., 2016). In more recent years, the increasing understanding of the key role of specifically lysosomal dysfunction in PD (Nalls et al., 2019) has highlighted *GBA1* as a crucial molecular player with direct implications for aSyn homeostasis. The gene *GBA1* is the most common PD risk factor and encodes for the lysosomal enzyme GCase (Sidransky et al., 2009). GCase is active in the acidic endolysosomal lumen, hydrolyzing glucose moieties from the glucolipids glucosylceramide (GluCer) and glucosylsphingosine (GluSph) (Do et al., 2019). Homozygous *GBA1* loss-of-function mutations lead to the lysosomal storage disorder Gaucher’s disease, characterized by accumulation of GluCer and GluSph within the lysosomes of macrophages. In some Gaucher patients, manifestations of parkinsonism and dementia, as well as Lewy pathology, have been described (Wong et al., 2004). Heterozygous *GBA1* mutations are encountered in 7–12% of PD patients (*GBA1*-PD) (Do et al., 2019) and shown to increase risk for motor symptom severity and dementia progression (Winder-Rhodes et al., 2013).

GCase function is strongly and reciprocally connected to aSyn homeostasis (Figure 3), but GCase has also emerged as a molecular convergence target of pathways associated with multiple genetic or idiopathic PD forms. A Gaucher’s disease mouse model expressing mutant GCase showed hippocampal aSyn aggregation and memory deficits, and phenotypes significantly improved by adeno-viral wild-type *GBA1* overexpression (Sardi et al., 2011). Generally, GCase deficiency is associated with higher intracellular aSyn levels, in neuronal models and iPSC-derived DAergic neurons from Gaucher’s disease and *GBA1*-PD patients (Mazzulli et al., 2011; Schondorf et al., 2014; Aflaki et al., 2016; Kim et al., 2018; Burbulla et al., 2019). In particular, the buildup of GluCer on endolysosomal membranes promotes the formation of pathological high molecular weight aSyn species, further converted to insoluble species (Mazzulli et al., 2011; Zunke et al., 2018). Moreover, increased GluCer was also shown to de-stabilize aSyn tetramers (Kim et al., 2018), which in contrast to oligomers are proposed to represent physiological multimeric forms less prone to aggregate (Bartels et al., 2011; Dettmer et al., 2015), although this is still controversial. GluCer-induced aSyn forms have also been directly associated with endogenous neurotoxicity, as well as decreased neuronal viability upon endocytosis from healthy DAergic neurons (Zunke et al., 2018). An equally important aspect of the aSyn–GCase interaction is that increased aSyn levels can *per se* impair lysosomal GCase activity *via* improper enzyme maturation and trafficking, an effect that also disrupts other lysosomal enzymes (Mazzulli et al., 2016a; Cuddy et al., 2019). Specifically, aSyn disrupts the association between the SNARE protein ykt6, implicated in ER–Golgi trafficking, and membranes, validated in iPSC-derived DAergic neurons from *SNCA* triplication- or A53T aSyn PD patients (Cuddy et al., 2019). Further mechanistic investigations in iPSC-derived DAergic neurons have revealed that some PD-associated proteins exert an inhibitory effect on GCase activity that can, at least partly, be attributed to DA oxidation. In long-term cultures of DJ-1-deficient iPSC-derived DAergic neurons, a time-dependent pathogenic cascade has been discovered that started with mitochondrial oxidative stress promoting the generation of DA-Qs (Burbulla et al., 2017). These species of oxidized DA led to disruption of GCase activity through modification of cysteine residues at the GCase catalytic center (Burbulla et al., 2017). A similar concurrent elevation of oxidized DA and reduced GCase activity was also observed in *PINK1*, *PARKIN*, *LRRK2*, and *SNCA* triplication iPSC-derived DAergic neurons (>90 days of neurons in culture), but also in sporadic PD iPSC-derived DAergic neurons at later time points (>180 days) (Burbulla et al., 2017). Further studies will be important to identify additional pathways affecting GCase activity in different PD forms beyond DA metabolism (Laperle et al., 2020).

## Disease-Modifying Therapies for PD

Thus far, there is strong evidence indicating that lysosomal dysfunction and mitochondrial oxidative stress are key factors for DAergic neuron vulnerability and tightly connected to aSyn homeostasis in PD etiopathology. Moreover, calcium and iron can have a significant contribution in pathways related to oxidative stress and pathological aSyn formation. Consequently, in this section, we discuss therapeutic approaches and clinical studies focusing on the molecular target GCase, as well as calcium and iron, as three important factors with high relevance for aSyn homeostasis. Notably, such therapeutic approaches are not directly targeting pathological aSyn, but could possibly restore the unbalanced equilibrium between aSyn, mitochondrial, and lysosomal pathways (for aSyn-targeted therapies, see Charvin et al., 2018).

The function of GCase is crucial for both glucolipid metabolism and aSyn homeostasis. Increasing GCase activity is, at present, the overarching aim of translational efforts specifically targeting PD lysosomal dysfunction. To this end, one major emerging approach in *GBA1*-PD is promoting mutant lysosomal GCase translocation to the lysosome *via* small molecule modulators, validated in iPSC-derived DAergic culture studies. For instance, long-term cultured (>100 days) iPSC-derived DAergic neurons from Gaucher patients exhibited key disease-related phenotypes of GluCer/GluSph and aSyn accumulation (Aflaki et al., 2016). Treatment with a GCase modulator (21 days, compound NCGC607) improved both phenotypes (Aflaki et al., 2016). Importantly, small molecule enhancement of GCase activity (8 days, compound NCGC00188758) could also reduce aSyn in *SNCA*- and *ATP13A2/PARK9*-PD iPSC-derived DAergic long-term cultured neurons (>90 days), which also showed reduced GCase activity (Mazzulli et al., 2016b). Alternatively, promoting wild-type GCase activity (10 days, compound S-181) successfully reduced glucolipids and DA/aSyn pathological phenotypes in *LRRK2*, *DJ-1*, and *PARKIN* as well as sporadic PD long-term iPSC-derived DAergic cultures (up to day 160) (Burbulla et al., 2019). Furthermore, application of a farnesyltransferase inhibitor (LNK-754) could also promote GCase lysosomal translocation by promoting the ykt6–Golgi membrane interaction, reducing pathological aSyn in *SNCA*-PD DAergic neurons (Cuddy et al., 2019). Indirect targeting of GCase activity includes pharmacological or genetic LRRK2 inhibition, assessed in *GBA1- and LRRK2-*PD iPSC-derived DAergic neurons (Nguyen and Krainc, 2018; Ysselstein et al., 2019). This approach could significantly improve the pathological accumulation of oxidized DA and aSyn (including pS129-aSyn) (Ysselstein et al., 2019). Currently, the two main approaches to increase GCase activity in *GBA1*-PD patients that reached clinical trials are either compounds aiming to enhance the activity of mutant GCase function or targeted gene therapy. The small molecule chaperone ambroxol has been successfully used to enhance mutant GCase activity and reduce pathological aSyn in *GBA1* and *SNCA* mouse models (Migdalska-Richards et al., 2016). Results from a Phase 2 clinical trial using ambroxol in a small patient cohort with and without *GBA1* mutations (NCT02941822) suggested that the compound penetrated the CSF and targeted GCase, leading to increased total aSyn levels in the cerebrospinal fluid (CSF) (Mullin et al., 2020). However, the mechanisms underlying the effect of GCase targeting on total aSyn in CSF are not clear. Concerning gene therapy, a Phase 1/2a addresses the AAV (adeno-associated virus)9-mediated delivery of wild-type *GBA1* into the central nervous system (NCT04127578). Biochemical assessments include GCase activity and GluCer/GluSph levels in the blood and CSF biofluids of *GBA1*-PD patients.

Voltage-gated calcium channels are targets for PD pharmacological interventions. The effects of isradipine, an L-type (Ca_v_1) channel antagonist of the dihydropyridine class, have been more extensively studied with promising *in vitro* and *in vivo* results (Leandrou et al., 2019). Moreover, epidemiological studies suggested that dihydropyridine use was associated with reduced PD risk (Ritz et al., 2010; Pasternak et al., 2012). However, results from the completion of a Phase 3 clinical study of 3-year duration, in previously untreated early PD patients (NCT02168842), indicated that isradipine did not slow the rate of clinical PD progression, as measured by UPDRS (Unified Parkinson’s Disease Rating Scale) scores (parts I–III, corresponding to mental function, daily living, and motor function) (Parkinson Study Group Steady-Pd Iii Investigators, 2020). It thus remains to be examined whether a highly selective antagonist of Ca_v_1.3 channels, linked to the pacemaking activity-induced oxidative stress in SNpc DAergic neurons (Guzman et al., 2010), may show disease-modifying potential. An additional challenge in objectively assessing efficacy of calcium-targeting approaches is the identification of specific target engagement markers.

Targeting iron in PD aims at alleviating DAergic neurons from an agent that can enhance oxidative stress and aSyn pathology. To this end, administration of the iron chelator deferiprone to mice prior to neurotoxic lesion was shown to protect them from nigrostriatal DAergic loss and mitigate oxidative stress (Devos et al., 2014). A 2-year pilot, double-blind, placebo-controlled randomized clinical trial using deferiprone in early stage PD (NCT00943748), in addition to DAergic therapy, indicated reduction of SN iron deposition and some improvement of motor symptoms based on UPDRS score (Devos et al., 2014). However, cessation of deferiprone treatment led to reappearance of SN iron deposition and waned clinical improvement (Devos et al., 2014). Moreover, PD patients with lower ceruloplasmin activity in the CSF showed a greater reduction of SN iron levels within a 1-year deferiprone regimen (Grolez et al., 2015). Therefore, efficacy of iron chelation may vary between individuals, due to underlying differences in iron metabolism in the periphery. A follow-up Phase 2 clinical trial for 9 months (NCT02655315) and an additional 9-month Phase 2 study specifically in early stage PD (NCT02728843) further assess deferiprone for motor and non-motor symptoms (Movement Disorder Society-UPDRS scores).

The diverse genetic background of PD patients places a variable burden on the aSyn, mitochondrial, and lysosomal pathways of DAergic susceptibility, likely contributing to the heterogeneous clinical presentation. This realization intensifies the need for specific and sensitive biomarkers allowing for distinguishing disease stages, thus optimizing patient stratification. Such biomarkers could improve the evaluation or possibly predict patient responses to different therapies in the endeavor for personalized medicine. Under this scope, a thorough understanding of interactions between DAergic vulnerability pathways, aging, and environmental and genetic factors (Figure 3) can be instrumental to emerging strategies involving implantation of iPSC-derived DAergic progenitors from autologous sources into PD patients (Schweitzer et al., 2020).

## Conclusion and Perspectives

Developments in the field of genetics have revealed a strong genetic component underlying familial and sporadic forms of PD. Mechanistic evidence has highlighted a deleterious feedback cycle whereby mitochondrial, lysosomal, and aSyn pathways converge and lead to DAergic susceptibility. In this feedback loop, calcium and iron can have a significant contribution as modulators exacerbating synaptic vulnerability. So far, the use of iPSC technology has helped to demonstrate that lysosomal dysfunction, accumulation of oxidized DA, and pathological aSyn represent shared iPSC-derived DAergic neuron phenotypes, despite the heterogeneity of PD genetic backgrounds. The concept of mechanistic convergence in DAergic vulnerability suggests that disease-modifying therapies require specific molecular targets able to restore equilibrium between individual pathways. Ideally, novel biomarkers reflecting the pathological status of PD-related pathways will optimize patient stratification for testing therapeutic responses and potentially increasing benefits for specific PD subpopulations. Furthermore, considering the wide use of DAergic medication for relieving PD motor symptoms, it will be crucial to investigate in detail DA metabolism and particularly DOPAL build-up mechanisms using iPSC-derived DAergic neurons. Such studies will provide novel insight into how DA release/metabolism equilibrium is altered in PD and open new venues for DA detoxification approaches, potentially as early interventions in PD.

iPSC-derived DAergic neurons used for *in vitro* modeling of PD represent a platform for investigating mechanisms of PD etiopathology and facilitate early phases of drug discovery. This technology, however, also presents limitations for studying PD biology, and several crucial questions remain to be elucidated. For example, interpretation and comparison of findings among different studies can be complicated by the fact that DAergic neuron cultures were obtained with variable purity and analyses were performed at different *in vitro* ages. Therefore, establishing conditions for culturing iPSC-derived DAergic neurons from different genetic backgrounds over longer periods would have to be standardized to accurately monitor the manifestation of different phenotypes including aSyn accumulation, lysosomal function, and oxidative stress. Moreover, iPSC-derived DAergic neuron cultures are especially suitable for studying cell-autonomous pathways of PD vulnerability. Yet, experimental disease modeling paradigms using additional cell types, including but not limited to glial cells, are required to better recapitulate the cell-to-cell interactions of the midbrain *milieu*. To this end, utilizing iPSC technology allowing for neuron–glia co-cultures as well as midbrain organoids may provide important answers. Patient-specific iPSC-derived models will serve as a resource for *in vitro* and *in vivo* disease modeling and drug screening, with the goal of testing neuroprotective compounds and developing therapeutic interventions.

## Author Contributions

GM, DK, and LFB wrote the manuscript. All authors contributed to the article and approved the submitted version.

## Conflict of Interest

DK was the Founder and Scientific Advisory Board Chair of Lysosomal Therapeutics Inc., and Vanqua Bio. DK serves on the scientific advisory boards of The Silverstein Foundation, Intellia Therapeutics, and Prevail Therapeutics and is a Venture Partner at OrbiMed. The remaining authors declare that the research was conducted in the absence of any commercial or financial relationships that could be construed as a potential conflict of interest.
